# Patient-mediated knowledge translation (PKT) interventions for clinical encounters: a systematic review

**DOI:** 10.1186/s13012-016-0389-3

**Published:** 2016-02-29

**Authors:** Anna R. Gagliardi, France Légaré, Melissa C. Brouwers, Fiona Webster, Elizabeth Badley, Sharon Straus

**Affiliations:** 1 University Health Network, Toronto, Canada; 2 Université Laval, Quebec City, Canada; 3 McMaster University, Hamilton, Canada; 4 University of Toronto, Toronto, Canada; 5 St. Michael’s Hospital, Toronto, Canada

**Keywords:** Decision-making, Patient engagement, Knowledge translation, Systematic review, Shared decision-making, Implementation science, Arthritis, Cancer

## Abstract

**Background:**

Patient-mediated knowledge translation (PKT) interventions engage patients in their own health care. Insight on which PKT interventions are effective is lacking. We sought to describe the type and impact of PKT interventions.

**Methods:**

We performed a systematic review of PKT interventions, defined as strategies that inform, educate and engage patients in their own health care. We searched MEDLINE, EMBASE and the Cochrane Library from 2005 to 2014 for English language studies that evaluated PKT interventions delivered immediately before, during or upon conclusion of clinical encounters to individual patients with arthritis or cancer. Data were extracted on study characteristics, PKT intervention (theory, content, delivery, duration, personnel, timing) and outcomes. Interventions were characterized by type of patient engagement (inform, activate, collaborate). We performed content analysis and reported summary statistics.

**Results:**

Of 694 retrieved studies, 16 were deemed eligible (5 arthritis, 11 cancer; 12 RCTs, 4 cohort studies; 7 low, 3 uncertain, 6 high risk of bias). PKT interventions included print material in 10 studies (brochures, booklets, variety of print material, list of websites), electronic material in 10 studies (video, computer program, website) and counselling in 2 studies. They were offered before, during and after consultation in 4, 1 and 4 studies, respectively; as single or multifaceted interventions in 10 and 6 studies, respectively; and by clinicians, health educators, researchers or volunteers in 4, 3, 5 and 1 study, respectively. Most interventions informed or activated patients. All studies achieved positive impact in one or more measures of patient knowledge, decision-making, communication and behaviour. This was true regardless of condition, PKT intervention, timing, personnel, type of engagement or delivery (single or multifaceted). No studies assessed patient harms, or interventions for providers to support PKT intervention delivery. Two studies evaluated the impact on providers of PKT interventions aimed at patients.

**Conclusions:**

Single interventions involving print material achieved beneficial outcomes as did more complex interventions. Few studies were eligible, and no studies evaluated patient harms, or provider outcomes. Further research is warranted to evaluate these PKT interventions in more patients, or patients with different conditions; different types of PKT interventions for patients and for providers; and potential harms associated with interventions.

**Electronic supplementary material:**

The online version of this article (doi:10.1186/s13012-016-0389-3) contains supplementary material, which is available to authorized users.

## Background

Despite considerable research and clear policies in many health care systems, evidence-based practice has yet to be widely adopted [1–3]. Part of the problem is insufficient consideration of the patient-clinician consultation, which lies at the heart of clinical practice. In clinical settings, the implementation of knowledge depends on the exchange of information between health care providers and patients, and research evidence is used to support decision-making [4]. To promote evidence-based practice, interventions targeting health professionals have been evaluated; however, most had a small or inconsistent impact on patient care and associated outcomes [5–8]. In contrast, interventions targeting patients (or consumers) appeared to have a moderate to large impact on care delivery and clinical outcomes and represent a promising means by which to achieve health care system improvement in a way that is responsive to patient needs and values [5, 9, 10]. Other research shows that interventions aimed at both patients and providers may be more effective than targeting one group alone [11, 12]. If we are serious about improving health system performance and population health outcomes, we need to change the way we study knowledge translation to support the role of patients in clinical decision-making [13].

‘Patient-mediated’ knowledge translation interventions (PKTs), defined broadly as strategies that engage patients in their own health care, stands to improve patient knowledge, relationship with provider, appropriateness of health service use, satisfaction with the care delivery experience, adherence to recommended treatment and other health behaviour and outcomes [14]. PKT is focused on individual health and health care and is distinguished from patient involvement in organization- or system-level quality improvement, governance or policy-making [15]. Although individual patient preferences for engagement may vary, it is important to consider PKT when planning and implementing health system innovations and improvements [16]. However, the ability to do so is predicated on an understanding of the PKT options available, and when and how to apply them, which is currently lacking.

There is no single agreed-upon or comprehensive taxonomy or framework that describes PKT. The Effective Practice and Organisation of Care (EPOC) taxonomy of behavioural interventions [17], and a subsequent version that was tailored by Mazza et al. [18] to describe interventions that had been used to implement guidelines, include few PKT, namely financial incentives, mechanisms for lodging complaints or suggestions, mechanisms for accessing treatment such as mail order pharmacies and participation in organizational governance. We conducted a systematic review that further elaborated on the PKT in the EPOC/Mazza taxonomy with print material, education, counselling, reminders and group interaction (support groups, social media) though this too was limited by the focus on interventions used to implement arthritis, diabetes, colorectal cancer and heart failure guidelines over a 10-year period [19]. The Expert Recommendations for Implementing Change initiative consolidated numerous existing taxonomies of implementation strategies with input from experts [20]. However, the overarching taxonomy included interventions targeting a variety of stakeholders and provided little detail about the type and characteristics of interventions targeting patients.

There are no systematic reviews that have focused on the types and impact of PKT that could be delivered during clinical encounters. PKT approaches likely need to be tailored for patients whose engagement and health care needs differ by condition. Arthritis is a long-term chronic condition featuring variability in care delivery and suboptimal outcomes [21, 22]. An international group of rheumatologists found little evidence-based guidance for the design of patient education interventions and consequently recommended that further research be carried out to generate tools that would support patient engagement [23]. In contrast, cancer, or at least its management, would be considered acute, although some cancers such as breast or prostate cancer are considered chronic in nature because they are managed over many years. Cancer care would also benefit from improvements in service delivery and outcomes [24]. In particular, research shows that patients with cancer prefer, but often do not assume, a shared or active role in decision-making [25]. Therefore, we sought to systematically review studies that described and evaluated PKT for arthritis or cancer patients in ambulatory settings immediately before, during or upon conclusion of clinical encounters (henceforth referred to as clinical encounters) that focused on discussions about treatment or management. More specifically, the purpose of this study was to identify and describe effective strategies for PKT during clinical encounters. This would build on previous work in which we compiled several empirical and conceptual sources to generate a framework inclusive of a variety of objectives, settings, types of PKT that differed by format, content and delivery and potential outcomes [26].

## Methods

### Knowledge synthesis design

A systematic review was conducted [27]. The Preferred Reporting Items for Systematic Reviews and Meta-Analyses (PRISMA) criteria guided the conduct and reporting of the review [28] (Additional file 1). Data were publicly available so institutional review board approval was not necessary. A protocol for this review was previously published [26]. The methods described here provide details about updates to that protocol. The original protocol, published in 2011, was based on a conceptual framework inclusive of a variety of PKT objectives, settings, format, content and delivery. When the systematic review was completed, several years had passed, necessitating an update. However, considerably more research on patient engagement had emerged, requiring reconsideration of the scope of the review. Hence, the focus of the review was narrowed to consider engagement in the context of clinical encounters, thereby expanding on a portion of the framework published in the original protocol.

### Scoping the literature

To plan for the full-scale review, a preliminary scan of relevant literature was undertaken by searching MEDLINE for ‘arthritis’ or ‘neoplasms’ and ‘patient education as topic’. The search results were used to gain an understanding of the available literature. Since publication of the protocol [26], which included all potential settings, research on PKT had increased substantially. To focus this review and enhance the feasibility and timeliness of completion, we chose to focus on PKT delivered during clinical encounters. Paired study investigators independently screened the search results to identify relevant studies that met preliminary criteria. This refined the scope of the review and contributed to further development of screening criteria.

### Eligibility criteria

Inclusion and exclusion criteria were generated based on the PICO framework (patients, intervention, comparison and outcomes). The scope was limited to studies involving adult patients with osteoarthritis or rheumatoid arthritis and, because the cancer literature was vast, patients with breast cancer or prostate cancer. These are prevalent conditions that represent a high burden of disease. Encounters were defined as discussions about treatment or management of a disease or condition in community or hospital office-based settings in which patients routinely see physicians. Inpatient care was excluded. Interventions could be delivered immediately before (e.g. question prompt lists, summaries), during (e.g. summaries, decision aids) or upon conclusion (e.g. self-monitoring guides or templates, summaries) of physician visits by the physician, by other individuals including nurses, receptionists, health coaches, social workers, pharmacists, physiotherapists or research staff or through audiovisual means including print material, videos, computer programs, tablet or telephone applications or the Internet for use following the consultation provided they were offered in the office setting. Since the focus was on the clinical encounter, interventions of interest were those directed to individuals that could be delivered in a single visit rather than requiring multiple sessions or meetings. Searches were limited to English language systematic reviews, randomized controlled trials, interrupted time series or observational cohort studies. To avoid duplication, systematic reviews were not eligible, but they were retrieved to identify and acquire addition eligible studies. The references of all eligible studies were scanned. Publications in the form of editorials, protocols, abstracts, proceedings or conceptual analyses were not eligible. Studies were not eligible if interventions were offered at the time of treatment, or in home, community or other settings that were not the ambulatory clinical encounter; focused on prevention or screening; the effectiveness of medical tests or procedures; participation in clinical trials; patient characteristics such as self-efficacy or general views about information needs; validation of instruments for evaluating outcomes; non-informational interventions such as exercise, rehabilitation or provision of medical equipment; or interventions for health professionals to promote or enable patient engagement alone (i.e. not combined with a PKT intervention).

### Search strategy and screening process

A comprehensive literature search was conducted by using several indexed sources. ARG and a trained research assistant conducted searches with input from a medical librarian. MEDLINE, EMBASE and the Cochrane Library were searched on September 9, 2014 from 2005 to 2014 inclusive. We limited the search to research published in the most recent decade to characterize patient-mediated interventions most likely to be applied currently. The search strategy applied to all three indexed sources (Additional file 2) was purposefully broad to be as inclusive as possible because the scoping exercise revealed that relevant articles on PKT were not consistently indexed. Searches in all databases were last updated on February 2, 2015 to ensure that eligible studies published in 2014 were captured. ARG and the research assistant independently screened titles and abstracts according to specified eligibility criteria. Rather than resolving selection differences, all those selected by at least one reviewer were retrieved since ultimate judgment about inclusion must often be reserved until the full text is examined. If more than one publication described a single study and each presented the same data, the most recent was included.

### Data extraction

A data extraction form was developed to collect information on study design, number and type of participants, intervention design including mention of theory used to design intervention, content, mode of delivery, duration, personnel delivering the intervention, timing (before, during or after consultation) and impact, including satisfaction or harms associated with the intervention, which referred to patient-related outcomes that were reported by studies. Given research findings which demonstrated that interventions targeting both patients and providers may be more effective than targeting one group alone [11, 12], we also extracted data on clinician or organizational interventions that were meant to prepare for or support the delivery of PKT interventions, or the impact of PKT interventions on clinicians or organizations. ARG and the research assistant independently pilot-tested the form on the same three articles and compared findings through two iterations at which time data extraction was congruent. The research assistant extracted data, which was independently checked by ARG.

### Quality assessment

The methodological quality of eligible studies was assessed with the Cochrane Collaboration Risk of Bias tool for randomized controlled trials (RCTs) and a modified Downs and Black Quality Assessment Tool for observational studies [29, 30]. The potential risk of bias was reported for each study.

### Data analysis

Summary statistics were used to describe the number of studies by topic, country, year of publication, study design, risk of bias, the number of studies employing single and multifaceted interventions and the number that employed theory in intervention design. Data could not be pooled due to heterogeneity in study design, interventions and measures of impact reported across eligible studies. To categorize the interventions used in eligible studies, we compiled a framework based on three categories of engagement described in other published research (inform, activate, collaborate) [15, 31, 32] and support for engagement in each of these three categories from a previously published meta-review that focused on self-management but offered a range of relevant supports for engagement that might be provided to patients during clinical encounters [33] (Table 1). Studies were tabulated by study objective, intervention, timing with respect to consultation and outcome and category of type of engagement and type of support from this compiled framework. This allowed for the enumeration of the strategies underlying interventions used in eligible arthritis and cancer studies and scanning of potential association in PKT employed with outcomes. All findings were captured in a conceptual framework. To do this, each unique instance was noted for the type of PKT intervention (component, timing of delivery, personnel), level of engagement, support and outcome.

**Table 1:** Characteristics of patient-mediated knowledge translation (PKT)

Type of engagement^a^	Type of support^b^	Examples
Inform Text-based information that provides patients with knowledge about their condition and an understanding of how to manage it	Condition and treatment	Information and evidence about the condition, prognosis, what to expect and its management
	Activities of daily living	Information and advice on how to undertake generic activities such as hygiene, dressing, preparing meals and transportation
	Lifestyle advice	Information and guidance on lifestyle behaviours that support disease management
Activate Text-based prompts or tools to prompt action for actively managing the condition and enhancing quality of life	Decision aids	Informational resources that help people consider the benefits and harms of treatment options
	Lifestyle monitoring	Reminders, diaries or other prompts to support adherence to medication or recommended lifestyle behaviours
	Action plans for condition	Guidance specific to medical condition, providing signs of worsening condition, how to self-adjust treatment and response if deterioration continues
	Physiological monitoring	Self-evaluation tools to log and monitor physiological measures for personal assessment and to share with clinicians
	Psychological strategies	Mechanisms for problem-solving, goal-setting, reframing and relaxation
Collaborate Text-based links, prompts or tools that lead to interaction and engagement	Communication with providers	Guidance and prompts to facilitate communication with health care professionals
	Available resources	Links to or contact details for organizations that offer information, psycho-social support or financial aid
	Social support	Links to or contact details for organizations that offer support, mentoring or socializing

^a^From Carmen [15], Grande [31], Coulter [32]

^b^Adapted from Taylor et al. [33]: patient medical care and equipment were removed from original framework

## Results

### Search results

**Fig. 1:**
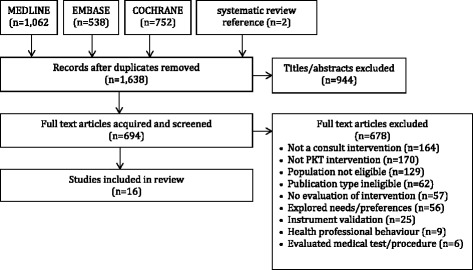
PRISMA diagram of eligible studies

The PRISMA diagram appears in Fig. 1. Data extracted from the 16 studies that were eligible for inclusion in the review appear in Additional file 3 [34–49].

### Characteristics of eligible studies

Five arthritis studies, published between 2007 and 2012, included 4 randomized controlled trials [38, 39, 45, 46] and 1 observational study with a before-after design [47]. Four were conducted in the USA and 1 in the UK. Of 4 randomized controlled trials, 1 had a low risk of bias and 3 had a high risk of bias. The single observational study had a low risk of bias. Eleven cancer studies, published between 2005 and 2013, included 8 randomized controlled trials [34–36, 40, 41, 43, 44, 48, 49] and 3 observational studies, 1 each with a prospective, comparative and before-after design [37, 42]. Six were conducted in the USA, and 1 each in Canada, China, France, Netherlands and the UK. Of 8 randomized controlled trials, 2, 3 and 3 had a low, unclear and high risk of bias, respectively. The three observational studies had a low risk of bias. Risk of bias scores for all studies are shown in Table 2.

**Table 2:** Characteristics and impact of patient-mediated knowledge translation (PKT) interventions used in eligible studies

Study ROB^a^	Objective (condition)	PKT			Patient outcome
		Intervention	Timing	Engagement (support)	
Single intervention					
Lam [35] L	Decision-making (cancer)	Booklet	After	Inform (condition)	+
				Activate (decision aid)	
vanTol Geerdink [36] U	Decision-making (cancer)	Brochure	After	Inform (condition)	+
				Activate (decision aid)	
Sivell [37] L	Decision-making (cancer)	Website	After	Inform (condition)	+
				Activate (decision aid)	
Jibaja Weiss [40] U	Decision-making (cancer)	Computer program	After	Activate (decision aid)	+/−
				Collaborate (communication)	
Lebret [42] L	Knowledge (cancer)	Print material	During	Inform (condition, lifestyle advice)	+
				Activate (lifestyle monitoring)	
Smith [43] L	Communicate and manage pain (cancer)	Counselling session	Before	Inform (condition)	+/−
				Activate (lifestyle monitoring)	
				Collaborate (communication)	
Walker [46] H	Knowledge (arthritis)	Brochure	After	Inform (condition)	+
Weng [47] L	Decision-making (arthritis)	Video	Before	Activate (decision aid)	+
Siminoff [48] H	Decision-making (cancer)	Computer program	During	Activate (decision aid)	+
Walker [49] H	Communication about treatment (cancer)	Video	Before	Collaborate (communication)	+
Multifaceted intervention					
Berry [34] U	Decision-making (cancer)	Brochure, list of websites, computer program	Before	Inform (condition)	+/−
				Activate (decision aid)	
				Collaborate (communication)	
deAchaval [38] H	Decision-making (arthritis)	Brochure, video	Before	Activate (decision aid)	+
McDonald [39] L	Communicate about pain (arthritis)	Information video, coaching video	Before	Inform (condition, lifestyle advice)	+
Kravitz [41] H	Communicate about pain (cancer)	Counselling session, print material	Before	Inform (condition)	+/−
				Collaborate (communication)	
Loiselle [44] L	Knowledge (cancer)	Computer program, list of websites	After	Inform (condition)	+
Franekel [45] H	Decision-making (arthritis)	Brochure, computer program	Before	Inform (condition)	+
				Activate (decision aid)	

+ All reported findings positive, +/− mixed findings

^a^Risk of bias: *H* high, *U* unclear, *L* low

### Conceptual framework

**Fig. 2:**
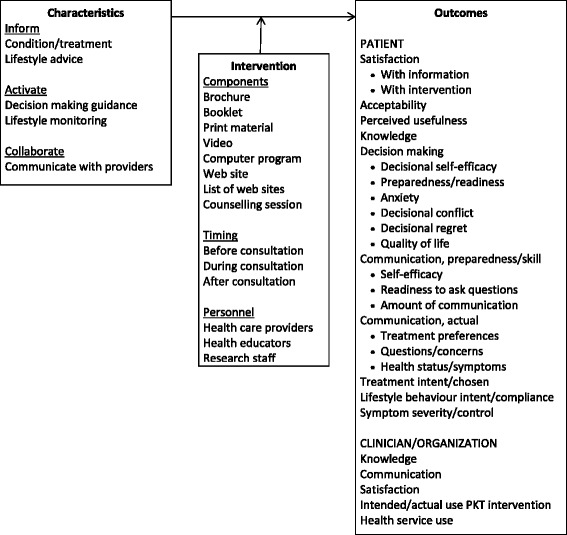
Conceptual framework of patient-mediated knowledge translation interventions for clinical encounters

Findings were captured in a conceptual framework of PKT interventions for clinical encounters (Fig. 2). The conceptual framework reflects, from among possible types of support for different types of engagement (Table 1), the types of support and engagement that were assessed in included studies and the outcomes reported in those studies. The framework includes different types of PKT interventions that varied by component or format of the intervention, timing with respect to when the consultation occurred and personnel who delivered the intervention. The PKT interventions were characterized based on the level of engagement and type of support. A range of impacts achieved in eligible studies are listed as outcomes, including impact on patients, and on clinicians or organizations. The findings are further discussed here.

### PKT interventions

No studies included interventions that were directed at clinicians or organizations to prompt or support delivery of patient-oriented PKT interventions. Table 2 shows the type of PKT intervention for patients used in each study including print material in 10 studies (brochures 5, booklets 1, variety of print material 2, list of websites 2), electronic material in 10 studies (video 4, computer program 5, website 1) and counselling in 2 studies. In 9 studies, the PKT intervention was meant to support decision-making. In these 9 studies, the PKT intervention was delivered before, during and after the consultation in 4, 1 and 4 studies, respectively, of which 3 were arthritis and six were cancer studies. Ten studies offered a single PKT intervention (2 arthritis, 8 cancer), and 6 studies offered a multifaceted PKT intervention (3 arthritis, 3 cancer). There did not appear to be any trend in the purpose of intervention, or type or timing of PKT intervention in single versus multifaceted interventions. Interventions were delivered by health care providers (physician, nurse) in 4 cancer studies, health educators in 3 cancer studies, research personnel in 3 arthritis studies and 2 cancer studies and a trained volunteer in 1 cancer study (not reported in 2 arthritis, 1 cancer study). One study referred to a formal theory that informed PKT design. A website to support decision-making about surgical treatment for breast cancer was based on the Theory of Planned Behaviour and Common Sense Model of Illness [37].

### Interventions and impact

#### Impact on patients

Table 2 shows that all studies achieved positive impact in one or more of the patient-level measures they reported. Heterogeneity of PKT interventions was apparent and precluded statistical pooling of the findings. Instead, based on observation, there did not appear to be trends in impact by the type of PKT intervention. For example, to support decision-making among 9 arthritis and cancer studies, single and multifaceted interventions were employed either before, during or after the consultation and they included a brochure, booklet, video, website, or computer program (single) or a combination of brochure and video; brochure and computer program; or brochure, computer program and list of websites (multifaceted). A range of measures were used to report various outcomes including knowledge, decision-making, communication and behaviour.

##### Knowledge

One RCT with a high risk of bias involving patients with rheumatoid arthritis found that both text and graphic information improved knowledge about arthritis and coping strategies, even among those with lower reading ability [46]. Knowledge about cancer and its treatment improved in one observational study with a low risk of bias in which the intervention was a computer application and a list of websites [44].

##### Decision-making

Decisional conflict was reduced or preparedness for decision-making improved in 2 RCTs and 1 observational study involving patients with osteoarthritis of the knee. One RCT with a high risk of bias provided information about treatment choices to patients in a 45-min videotape plus an information booklet [38], another RCT with a high risk of bias provided information via an interactive computer program that elicited values and preferences [45], and an observational study with a low risk of bias provided information in a 45-min videotape featuring patient narratives [47]. Decisional conflict, readiness or intention, anxiety and satisfaction with respect to treatment choice were improved in 6 cancer studies including five RCTs (1 low, 3 unclear, 1 high risk of bias) and 1 observational study with a low risk of bias [34–37, 40, 48].

##### Communication

An RCT with a low risk of bias reported no difference between the control and intervention groups in the amount of pain communicated by patients with osteoarthritis who first watched a 3-min informational video followed by a videotape featuring either a real or virtual health professional who encouraged them to practice communicating pain information aloud [39]. Two cancer randomized controlled trials, one with low and one with high risk of bias, improved pain communication with provider, although there was no difference in pain control self-efficacy, perceived barriers to pain control, pain severity or quality of life between the control and interventions groups [41, 43]. In one cancer randomized controlled trial with a high risk of bias, information about treatment planning visits and advice on questions to ask resulted in higher information preparedness and readiness to ask questions in the intervention group compared with the control group [49].

##### Behaviour

In one observational study with a low risk of bias that provided lifestyle advice to combat the side effects of androgen deprivation therapy for prostate cancer, most participants said they intended to or were already following the advice [42].

##### Satisfaction/harms

No studies assessed harms associated with interventions. Satisfaction with, or perceived usefulness, acceptability or ease of use of the intervention was assessed in 7 (43.8 %) studies including 2 arthritis [45, 47] and 5 cancer studies [34, 42, 44, 48, 49]. In all 7 studies, the majority of participants expressed favourable views of the intervention. In 3 cancer studies, there was no significant difference in satisfaction or perceived ease of use between the experimental and control groups [44, 48, 49]. In 1 cancer study, satisfaction with the information received decreased between the first and second visit [42]. In 1 arthritis study, 32 % thought that the decision-making tool did not at all or fully reflect their values [45], and in another arthritis study, 12 % thought that the information provided to support decision-making about surgery was not balanced [47].

#### Impact on clinicians/organizations

Two of 16 studies evaluated the impact of patient-oriented PKT interventions on clinicians or organizations. In 1 study, 91 urologists who provided print material to patients about androgen deprivation therapy were surveyed before and after the intervention period [42]. Overall satisfaction with the toolkit was high (82 %), and perceived benefits of the toolkit included improved dialogue with patients (62 %), follow-up (55 %), explanation of side effects (51 %), knowledge of guidance to be delivered to patients (30 %) and presentation of guidance to prevent side effects (13 %). However, 14 urologists thought the toolkit was not tailored to individual patients, too long or tedious or did not meet a need. Before the intervention, 92 % of urologists planned to give the toolkit to patients; this fell to 64 % upon study completion. In another study in which 250 patients newly diagnosed with breast or prostate cancer were provided with information on a CD-ROM and a list of websites, the impact on health service use was evaluated [44]. Being in the intervention group had no significant effect on health service use including number of visits to the oncologist (*p* = 0.51), time spent with the oncologist (*p* = 0.10) or time spent in telephone consultations (*p* = 0.56), but women spent more time with nurses (*p* = 0.03).

### Interventions characteristics

Table 2 summarizes the type of engagement and support underlying PKT interventions. There were no trends in PKT interventions characterized according to these characteristics and impact overall, or by type of outcome. For example, among the 9 arthritis and cancer studies that sought to improve decision-making, in three studies, the intervention was designed to activate patients by supporting the decision-making process; in five studies, the intervention was designed to both inform the patient about their condition and activate patients by supporting the decision-making process; and the intervention in one study was designed to inform and activate patients, and promote collaboration with providers by also offering advice on how to communicate issues of concern. Table 3 summarizes the level of engagement and type of support that formed the basis of PKT interventions. Most studies informed patients about their condition and its treatment, or activated patients with decision aids. Table 3 also reveals gaps where specific types of support were not used.

**Table 3:** Summary of PKT interventions and characteristics used in eligible studies

Characteristics		PKT intervention	Condition (reference)	
Engagement	Support		Arthritis	Cancer
			*n* = 5	*n* = 11
Inform	Condition and treatment	Brochure	46	34, 36
		Booklet	–	35
		Video	39	−
		Computer program	45	34, 44
		Website	−	37
		List of websites	−	34, 44
		Print material	−	42
		Counselling	−	41, 43
	Activities of daily living	−	−	−
	Lifestyle advice	Video	39	−
		Print material	−	42
Activate	Decision aids	Video	38, 47	−
		Brochure	38, 45	34, 36
		Booklet	−	35
		Computer program	45	34, 40, 48
		Website	−	37
		List of websites	−	34
	Lifestyle monitoring	Print material	−	42
		Counselling	−	43
	Action plans for condition	−	−	−
	Physiological monitoring	−	−	−
	Psychological strategies	−	−	−
Collaborate	Communicate with providers	Video	−	49
		Brochure	−	34
		Print material	−	41
		Computer program	−	34, 40
		Counselling	−	41, 43
	Available resources	−	−	−
	Social support	−	−	−

## Discussion

To the best of our knowledge, this study is among the first to describe the type of PKT interventions that could be employed in routine clinical encounters with arthritis or cancer patients during which treatment or management were discussed, their impact and the characteristics of PKT interventions associated with effectiveness. We observed that, while eligible studies were few, 9 of 16 had an unclear or high risk of bias, and only one employed theory in the intervention design, all achieved a positive impact on at least one of the outcomes measured. No studies specifically measured or reported patient harms associated with the intervention. We also observed that, although arthritis and cancer differ with respect to modes of treatment and types of clinical outcomes, and PKT interventions were investigated in more cancer studies, the types of PKT interventions were similar for both arthritis and cancer patients. No studies included interventions for clinicians or organizations as a means of prompting or supporting the delivery of PKT interventions, and 2 of 16 studies reported the impact of PKT interventions on clinicians or organizations.

Overall, few studies were eligible. Other systematic reviews investigating patient-provider interaction also found few eligible studies so this finding is not surprising [11, 12]. However, it is notable given the recognized potential for patient activation and engagement to improve health care outcomes and the need for strategies to support both patients and providers to usher in the ‘patient engagement era’ [50]. Many have advocated for the development and implementation of knowledge-based tools at the point-of-care to better inform, educate and engage patients [51–54]. Research shows that a variety of types of informational tools directed at patients can achieve numerous beneficial outcomes [33, 55–59]. In other research, we found that informational tools for patients with colorectal cancer, diabetes and heart disease also achieved positive outcomes [60]. Given the need for such resources [61, 62], using a modified Delphi process and interviews with international experts in guideline development and implementation, we generated criteria, methods and considerations for developing informational tools [63, 64].

While all studies demonstrated beneficial outcomes for patients, the interventions did not perform flawlessly. For example, in 1 cancer study, satisfaction with the information received decreased over time, and in 2 arthritis studies, some participants thought that the information was not consistent with their values or not balanced. It is also notable that, in 3 controlled or uncontrolled comparative studies, there was no difference in satisfaction or perceived usefulness of print material such as brochures compared with electronic information or more complex decision aids. The heterogeneity of interventions employed in eligible studies warrants some discussion. In part, this was because 10 of 16 eligible studies employed a single intervention, and all achieved a positive impact as did the 6 studies that employed multifaceted interventions. The need for single versus multifaceted interventions has been a source of debate. A recent meta-review of 25 systematic reviews that compared direct and indirect effect size and dose-response of single and multifaceted strategies showed no benefit of multifaceted over single strategies [65]. In part, this was because interventions varied. Intervention components could be broadly categorized as print material (brochure, booklet, variety of print resources, list of websites), digital material (video, computer program, website) or brief educational counselling, or some combination of these. Notably, counselling was used in only two studies, thus informational tools, either static (print) or more visual or interactive (electronic) predominated. By analyzing the interventions employed with a conceptual framework of PKT characteristics, this study revealed several types of interventions for engaging patients that were not employed (gaps summarized in Table 3). For example, no studies evaluated interventions meant to inform patients about activities of daily living; interventions meant to activate patients through action plans, physiological self-monitoring instructions or advice about psychological strategies; or interventions meant to promote collaboration by providing information about available social support or other resources. Therefore, in ongoing research, systematic reviews could be conducted specifically to investigate the design and impact of offering those strategies before, during or following clinical encounters. If few or none were available, then primary research would be warranted.

Despite evidence that PKT is best achieved by targeting patients and providers [11, 12], no studies in this review included interventions for clinicians or organizations, and only 2 of 16 studies assessed the impact of PKT interventions on clinicians or organizations. These findings too were largely positive. For example, in one study, most physicians perceived the PKT intervention as useful [42], and in another study, there were no unintended consequences on health service use [44]. Physicians have expressed challenges they face when involving patients in decision-making such as reluctance to give up traditional decision-making roles, lack of time or training in communication and little organizational support to help them engage patients [50, 51, 66]. Thus, interventions are needed among clinicians and organizations to embrace and adopt PKT. In this regard, further investigation is needed on the feasibility of delivering PKT interventions during clinical encounters. This review found that most studies offered interventions before (8 of 16) or upon conclusion (6 of 16) of consultations, and they were delivered by researchers or health educators engaged for the study (9 of 13), or nurses or physicians (4 of 13) in studies that reported personnel. If PKT interventions, which appear to achieve beneficial outcomes, are to be routinely offered, then health care delivery systems or institutions will need to recognize the need for these roles and allocate resources for such personnel so that the additional workload is not the sole responsibility of individual providers. Perhaps research in this regard could employ the recently published Measuring Organisational Readiness for Patient Engagement (MORE) scale as a means of assessing feasibility [67].

One study in this review employed theories or models to inform and optimize the design of PKT interventions [37]. Based on study findings, we modified a preliminary conceptual framework that we had previously developed [26] with types of engagement and support, types of interventions and associated outcomes that emerged from included studies. This generated a more refined version that was validated by applying it against studies specific to arthritis or cancer and for interventions that could be implemented one time in the context of routine clinical encounters. This version of the conceptual framework, which identifies options for the design of PKT interventions that have achieved positive outcomes, can be used by health care policy-makers, managers and providers to plan and implement PKT interventions that are tailored for desired types of engagement and support. This conceptual framework recognizes more patient-specific options for PKT than previous taxonomies of behaviour-change interventions [18–20]. It is also distinct from the work of Colquhoun et al. who blended numerous existing taxonomies of intervention stakeholders, components and modes of delivery with theory of causal mechanisms derived from the Behaviour Change Wheel [68]. Our research builds on and advances the work of Colquhoun by focusing on interventions targeted to patients, in particular those with arthritis or cancer, and identifying potential outcomes that may be achieved by using particular types of interventions for different types of engagement, although this remains to be confirmed through further research.

Several issues may limit the interpretation and use of these findings. Our review focused on arthritis and cancer and did not cover other clinical domains. Few studies were eligible, perhaps due to stringent eligibility criteria. Notably, 129 studies were excluded because they focused on special populations (i.e. disabled, pregnant), or cancer patients in general rather than focusing on breast or prostate cancer patients. Among the included studies, just over half had an unclear or high risk of bias, so the results must be interpreted with some caution. As a result, this review did not reveal links between types and characteristics of PKT and specific outcomes. Therefore, further research is needed to apply the conceptual framework generated here against studies that employ interventions during routine clinical encounters for patients with other forms of cancer or other acute conditions to assess the framework’s generalizability. The findings may not be transferrable to PKT interventions delivered in home, community or other settings that were not the ambulatory clinical encounter. Although we searched standard indexed sources of published medical literature, the search strategy may not have identified all relevant studies. We did not search the grey literature, assuming that most empirical research on PKT interventions would be found in indexed databases. Publication bias, or the tendency for journals to publish positive results, may have influenced the number and type of studies that were retrieved. However, we employed rigorous methodology that complied with standards for the conduct and reporting of systematic reviews [28] and used a unique conceptual framework to describe the features of PKT interventions, thereby providing insight on the design of a range of options that could be employed by others. Furthermore, our study was distinct from other systematic reviews that also demonstrated the positive impact of patient-oriented knowledge-based interventions [32, 33, 55–59] because it characterized interventions based on the conceptual framework. In doing so, we identified a range of PKT interventions that resulted in beneficial outcomes for patients with acute and chronic conditions in the context of clinical encounters to discuss treatment or management options and characterized the interventions to show the need for PKT interventions to inform and activate patients.

## Conclusions

This systematic review found that a variety of PKT interventions offered immediately before, during or upon conclusion of clinical encounters that focused on discussion about treatment or management achieved a positive impact on at least one or more of the outcomes measured including satisfaction, knowledge, decision-making, communication and behaviour. This was true regardless of condition (arthritis, breast or prostate cancer), PKT intervention format, timing with respect to the encounter, personnel who delivered the intervention, level of engagement, type of support or delivery as a single or multifaceted intervention. No studies assessed patient harms associated with interventions, and no studies included interventions to prompt or support PKT intervention delivery by clinicians or organizations. One study found that clinicians perceived the PKT intervention as useful, and another reported no unintended consequences on health service use. Eligible studies were few, and most employed PKT interventions that informed patients about their condition and its treatment or management, or activated patients with decision aids. Therefore, further research is warranted to evaluate these PKT interventions in more patients or patients with different conditions; different types of PKT interventions for patients and for clinicians and organizations; and potential harms associated with interventions for patients, clinicians and organizations. The conceptual framework generated by this research can be used by others to plan, implement and evaluate PKT interventions.

## Availability of supporting data

The data set(s) supporting the results of this article is(are) included within the article and its additional file(s).

## Additional files

Additional file 1: PRISMA reporting criteria. (DOC 59 kb)

Additional file 2: Search strategy used in MEDLINE. (DOCX 14 kb)

Additional file 3: Data extracted from eligible studies. (DOCX 27 kb)
