# Supplementary material for: Patient-mediated knowledge translation (PKT) interventions for clinical encounters: a systematic review
Source: Implement Sci. 2016 Feb 29;11:26. doi: 10.1186/s13012-016-0389-3 (PMC4770686; doi:10.1186/s13012-016-0389-3)
Supplement: Supplementary file 2 — Search strategy used in MEDLINE. (DOCX 14 kb) [file 13012_2016_389_MOESM2_ESM.docx]

Additional File 2. Search strategy used in MEDLINE

| Search | Terms/Commands |
| --- | --- |
| 1 | Patient Education as Topic/ |
| 2 | Patient Participation/ |
| 3 | Self care/ |
| 4 | 1 or 2 or 3 |
| 5 | exp Arthritis/ or breast neoplasms/ or prostate neoplasms/ |
| 6 | 4 and 5 |
| 7 | limit 6 to (english language and humans and yr=2005 to 2014 and "all adult (19 plus years)") |
| 8 | limit 7 to (controlled clinical trial or meta analysis or multicenter study or randomized controlled trial or systematic review) |
| 9 | qualitative research/ or focus groups/ or interviews as topic/ or health care surveys/ or questionnaires.mp. |
| 10 | 7 and 9 |
| 11 | 8 not 10 |
| 12 | limit 11 to (addresses or comment or editorial or interview or lectures or letter or news) |
| 13 | 11 not 12 |
