# Supplementary material for: Patient-mediated knowledge translation (PKT) interventions for clinical encounters: a systematic review
Source: Implement Sci. 2016 Feb 29;11:26. doi: 10.1186/s13012-016-0389-3 (PMC4770686; doi:10.1186/s13012-016-0389-3)
Supplement: Supplementary file 3 — Data extracted from eligible studies. (DOCX 27 kb) [file 13012_2016_389_MOESM3_ESM.docx]

Additional File 3. Data extracted from eligible studies

| Study  Country  Risk of bias | Study Design  Participants | Intervention Details | Impact on patients, clinicians or organizations |
| --- | --- | --- | --- |
| Berry  2013 [34]  United States  unclear | RCT of 494 men with prostate cancer recruited from 6 clinics in 4 cities | Patient  Content: information about prostate cancer treatment options and advice on how to share issues of concern with physician  Mode: educational brochure, links to web sites (control); brochure, web sites plus computer decision support program (intervention)  Duration: NR  Participants (follow-up/recruited): Intervention (233/266), Control (203/228)  Personnel: NR  Theory: none  Timing: before consultation  Clinician/Organization  none | Patient  Decisional conflict significantly improved in intervention group for uncertainty (95% CI, 7.01 to 0.22, p=0.04) and values clarity (95% CI, 5.85 to 1.30, p=0.002) but not for informed, support and effective decision subscales. The intervention scored high for acceptability (mean 25.1, SD 3.8) and value of information (mean 3.8, SD 1.0). No harms were assessed.  Clinician/Organization  none |
| Lam  2013 [35]  China  low | RCT of 276 women with newly diagnosed early-stage breast cancer recruited from 2 clinics in Hong Kong | Patient  Content: summary of clinical guideline on surgical management options for early stage breast cancer, differences between options, benefits and risks as described by women having had surgery, personal worksheet to facilitate values clarification, and structured guidance to achieve a decision  Mode: discussion with physician (control), booklet (intervention)  Duration: NR  Participants (follow-up/recruited): Intervention (113/138), Control (112/138)  Personnel: Nurse  Theory: none  Timing: after consultation  Clinician/Organization  none | Patient  Choice of surgery and anxiety scores did not differ between groups. Control patients reported significantly greater decisional conflict 1 week after consultation (p=0.016), and decisional regret (p=0.026) and depression scores (p=0.001) at 10 months after surgery. Satisfaction or harms were not assessed.  Clinician/Organization  none |
| van Tol-Geerdink 2013 [36]  Netherlands  unclear | RCT of 240 patients newly diagnosed with prostate cancer recruited from 3 hospitals | Patient  Content: Information about the process, risk and benefits of surgery or radiotherapy  Mode: discussion with physician (control), brochure (intervention)  Duration: NR  Participants (follow-up/recruited): Intervention (163/163), Control (77/77)  Personnel: Researcher  Theory: none  Timing: after consultation  Clinician/Organization  none | Patient  Intervention group preferred brachytherapy more frequently (20% vs 8%, p=0.02) and remained undecided less frequently (2% vs 8%, p<0.05) than control group. Satisfaction or harms were not assessed.  Clinician/Organization  none |
| Sivell  2012 [37]  United Kingdom  Low | Observational study of 144 women newly diagnosed with breast cancer recruited from one clinic in 3 cities | Patient  Content: information on breast cancer, surgical treatment options, risks and benefits, recovery period, psychosocial and cosmetic outcomes, possible further treatment, video clips of health professionals and of patients , structured decision making guidance (treatment intentions, preferences, views of significant others)  Mode: web site  Duration: mean 24 min, median 21 min, range 55 seconds to 1 hour, 27 minutes  Participants (follow-up/recruited): 54/144  Personnel: Specialist breast care nurses  Theory: Theory of Planned Behavior, Common Sense Model of Illness Representations  Timing: after consultation  Clinician/Organization  none | Patient  Over 80% of participants visited BresDex once or twice. Readiness to make a decision increased significantly from baseline (F 637.31, p<0.001). Knowledge and intention to choose breast conserving surgery increased, but not significantly. Satisfaction or harms were not assessed.  Clinician/Organization  none |
| de Achaval 2012 [38]  United States  high | RCT of 211 patients with OA knee pain recruited from the community and hospital in a single city | Patient  Content: treatment choices and implications for knee OA including medical and surgical management  Mode: print booklet of treatment choices (control); video decision aid plus booklet (group 1); video decision aid plus booklet plus interactive computer program to elicit values and preferences (group 2)  Duration: print booklet 20 minutes; video 45 minutes; interactive program 15 minutes  Participants (follow-up/recruited): group 1 (70/70), group 2 (69/70), control (69/71)  Personnel: study coordinator  Theory: none  Timing: before consultation  Clinician/Organization  none | Patient  Decisional conflict decreased significantly in all groups (p<0.05). Group 1 achieved the greatest reduction compared with Control and Group 2 (p<0.001). Satisfaction or harms were not assessed.  Clinician/Organization  none |
| McDonald 2011 [39]  United States  low | RCT of 30 patients with OA knee pain aged 60 or older from two independent living homes in a single city | Patient  Content: OA pain information including type, quality, source, location, intensity, duration, effect on personal lifestyle, functional status, current pain treatments, weight management and exercise regimen  Mode: All participants watched a pain communication video (control), then watched either a videotaped health professional or virtual health professional encourage them to practice communicating their pain and to talk about it with their physician (intervention)  Duration: pain communication video 3 minutes  Participants (follow-up/recruited): no coach (11/1), virtual coach (12/12), video coach (7/7)  Personnel: administered by research assistant; all videos were female health professionals  Theory: none  Timing: before consultation  Clinician/Organization  none | Patient  Amount of pain communicated was not significantly different between groups: virtual (mean 6.3, SD 3.17), video (mean 3.0, SD 2.08), no coach (mean 5.2, SD 2.40) (p=0.06). Satisfaction or harms were not assessed.  Clinician/Organization  none |
| Jibaja-Weiss 2011 [40]  United States  unclear | RCT of 100 lower literate and multi-ethnic women with newly diagnosed early stage breast cancer recruited from 2 hospitals | Patient  Content: video scenarios and interactive modules for values clarification exercise, recording of concerns, deliberation over treatment options, and advice for communicating with physician  Mode: print material (control), computer program (intervention)  Duration: NR  Participants (follow-up/recruited): Intervention (40/51), Control (36/49)  Personnel: Case manager  Theory: none  Timing: after consultation  Clinician/Organization  none | Patient  Intervention patients were more likely to prefer modified radical mastectomy (p=0.018) and had significantly improved knowledge before surgery compared with control patients (p<0.001). Decisional conflict was similar between groups. Satisfaction or harms were not assessed.  Clinician/Organization  none |
| Kravitz  2011 [41]  United States  high | RCT of 307 cancer patients (56.3% breast cancer) reporting moderate to severe pain recruited from 3 health systems  and 1 private practice in one city | Patient  Content: assess individual learning needs, goals and values, provide information and skill-building exercises to increase self-efficacy and prompt patient-physician communication, and rehearse new skills  Mode: print material and one-on-one (no rehearsal for control) Duration: 20-40 min  Participants (follow-up/recruited): Intervention (126/157), Control (132/150)  Personnel: Health educator  Theory: none  Timing: before consultation  Clinician/Organization  none | Patient  Communication self-efficacy (p<0.001), pain-related impairment (p=0.01) improved significantly more for intervention group. No difference in pain control self-efficacy, pain severity or pain misconceptions between groups. Satisfaction or harms were not assessed.  Clinician/Organization  none |
| Lebret  2010 [42]  France  low | Observational study of 585 men with prostate cancer about to receive androgen deprivation therapy in 91 urology clinics | Patient  Content: information about therapy, effects on body, advice on diet, exercise to reduce side effects, recipe booklet, lifestyle diary  Mode: print material  Duration: 25 min  Participants (follow-up/recruited): 287/585  Personnel: Urologists  Theory: none  Timing: during consultation  Clinician/Organization  none | Patient  Among respondents (287/585) 82% held positive views of the toolkit; 83% said they read it; 93% said they were following the advice; 36% planned to follow the advice. After the first visit 82% were glad or very glad that they were given the toolkit; after the second visit 76% were fairly or highly satisfied with the toolkit while 19% were not really or not at all satisfied. No harms were assessed.  Clinician/Organization  Overall satisfaction among 91 urologists was 82%. Perceived benefits were improved dialogue with patients (62%), follow-up (55%), explanation of side effects (51%), knowledge of guidance to be delivered to patients (30%) and presentation of guidance to prevent side effects (13%); 15.4% of urologists thought the toolkit was not tailored to individual patients, too long or tedious, or did not meet a need. Before the intervention 92% of urologists planned to give the toolkit to patients, this fell to 64% upon study completion. |
| Smith  2010 [43]  United States  low | RCT of 89 patients with breast cancer reporting moderate to severe persistent pain recruited from 4 teaching and 1 private hospital in one city | Patient  Content: information about pain, pain medication, how to communicate pain to providers and monitor pain with a diary; role play of communication and diary use  Mode: nutrition session (control), training session (intervention)  Duration: 30 min  Participants (follow-up/recruited): Intervention (37/47), Control (37/42)  Personnel: psychologists, educators or public health personnel  Theory: none  Timing: before consultation  Clinician/Organization  none | Patient  Intervention group reported significantly lower barriers score compared with control group (p=0.04). No statistically significant difference between groups in pain relief, pain management, quality of life or distress. Satisfaction or harms were not assessed.  Clinician/Organization  none |
| Loiselle  2009 [44]  Canada  low | Observational study of 250 patients with newly diagnosed breast or prostate cancer recruited from 4 clinics in 1 hospital | Patient  Content: information on anatomy, treatment, psychosocial oncology, list of web sites with information about breast or prostate cancer  Mode: usual discussion with physician or brochure (control), CD-ROM, list of web sites (intervention)  Duration: 1 hour  Participants (follow-up/recruited): Intervention (129/148), Control (101/102)  Personnel: Trained volunteer or the study medical librarian  Theory: none  Timing: after consultation  Clinician/Organization  none | Patient  Intervention group reported significantly greater satisfaction with cancer information (p<0.01) compared with control group. At T1 63.3% were very satisfied with the information they received from their health care professional; this was significantly greater among the control group (78.4%, p<0.001). At T2 significantly greater satisfaction reported by the experimental group (p<0.01). There was no significant difference in satisfaction between groups at T3. No harms were assessed.  Clinician/Organization  Being in the intervention group had no significant effect on health service use including number of visits to the oncologist (p=0.51), time spent with the oncologist (p=0.10) or time spent in telephone consultations (p=0.56), but women spent more time with nurses (p=0.03) |
| Fraenkel 2007 [45]  United States  high | RCT of 87 veterans with OA knee pain aged 60 or older from a single primary care outpatient clinic | Patient  Content: Definition, risk factors, symptoms and treatment of OA (pamphlet); tradeoffs between treatment characteristics of route of administration, likelihood of expected benefit and risk of adverse effects (computer program)  Mode: Two-page pamphlet (control), interactive computer program to elicit values and preferences (intervention)  Duration: NR  Participants (follow-up/recruited): intervention (44/47), control (40/40)  Personnel: Facilitated by research assistant  Theory: none  Timing: before consultation  Clinician/Organization  none | Patient  Decisional self-efficacy (p=0.0004), preparedness to participate in decision-making (p=0.0001) and arthritis self-efficacy (p=0.05) were greater in the intervention group. 98% of participants thought that the decision making tool was easy or very easy to use; 86% would recommend it to other patients with knee pain; 68% felt that it reflected their values, 27% felt that it somewhat reflected their values; and 5% felt that it reflected their values a little or not at all. No harms were assessed.  Clinician/Organization  none |
| Walker  2007 [46]  United Kingdom  high | RCT of 363 RA patients from 3 hospitals in a single city | Patient  Content: Information about arthritis, coping strategies and answers to common questions  Mode: Print booklet (control), or print booklet plus print “mind map” of information presented diagrammatically with key words and images (intervention)  Duration: Provided after seeing rheumatologist so that patients can review at home  Participants (follow-up/recruited): intervention (175/175), control (188/188)  Personnel: NR  Theory: none  Timing: after consultation  Clinician/Organization  none | Patient  There was a significant increase in knowledge in both groups from baseline but no significant difference between groups (-0.91, p>0.3). Poor readers in both groups were similarly less likely to improve knowledge. Satisfaction or harms were not assessed.  Clinician/Organization  none |
| Weng  2007 [47]  United States  low | Observational study of 64 patients with OA of the knee aged 55 or older from several primary care clinics in one city | Patient  Content: Pathogenesis and treatment options for OA plus patient interviews about why they chose their particular treatment (surgical versus medical)  Mode: Videotape  Duration: 45 minutes  Participants (follow-up/recruited): 64/102  Personnel: NR  Theory: none  Timing: before consultation  Clinician/Organization  none | Patient  Mean decision conflict score for the entire group improved from 39.4 to 25.8 (p=0.001). African American postoperative mean expectations improved from 41 to 31 for pain (p=0.04) and from 38 to 30 for physical function (p=0.09) while Caucasian mean expectations remained largely unchanged. The majority of patients agreed or strongly agreed that the video was informative, easy to understand, and would be helpful in deciding about surgery; 88 patients reported that they thought the video was balanced; 14 thought it favoured nonsurgical intervention and 5 thought it favoured surgery. No harms were assessed.  Clinician/Organization  none |
| Siminoff 2006 [48]  United States  high | RCT of 432 women that had surgical treatment for breast cancer recruited from 14 practices in 2 cities | Patient  Content: information on risk of death from cancer and other causes after surgery only versus surgery plus chemotherapy or other treatment to inform decisions about adjuvant therapy  Mode: brochure (control), computer program (intervention)  Duration: NR  Participants (follow-up/recruited): Intervention (234), Control (171)  Personnel: Health educator  Theory: none  Timing: during consultation  Clinician/Organization  none | Patient  Intervention group was less likely to choose adjuvant therapy compared with the control group (OR 0.32, p=0.02), and rated the decision guide more influential than those in the control group (p<0.001). Most patients in both the decision guide (94.1%) and pamphlet group (97.0%, p=0.4) thought they were easy to understand; 54.5% of decision guide patients thought it was very helpful compared with 34.4% of pamphlet patients (p<0.001). No harms were assessed.  Clinician/Organization  none |
| Walker  2005 [49]  United States  high | RCT of 95 patients diagnosed with breast cancer recruited from 1 centre | Patient  Content: portrayal of a typical treatment planning visit, advice on questions to ask  Mode: brochure (control), video (intervention)  Duration: 19 min  Participants (follow-up/recruited): Intervention (42/48), Control (37/47)  Personnel: Research assistant  Theory: none  Timing: before consultation  Clinician/Organization  none | Patient  Intervention group scored significantly higher for quality of life (p=0.048), optimism (0.042), satisfaction with intervention (p=0.049), information preparedness (p=0.05) and readiness to ask questions (p=0.05) compared with control group. There was no significant difference in satisfaction between the experimental videotape and control pamphlet groups (p=0.042); minorities were significantly more satisfied in the video group compared with minorities in the pamphlet group (p=0.007). No harms were assessed.  Clinician/Organization  none |

OA osteoarthritis, RA rheumatoid arthritis, NR not reported
